# C-Reactive Protein as a Prognostic Indicator in COVID-19 Patients

**DOI:** 10.1155/2021/5557582

**Published:** 2021-04-29

**Authors:** Mahmoud Sadeghi-Haddad-Zavareh, Masomeh Bayani, Mehran Shokri, Soheil Ebrahimpour, Arefeh Babazadeh, Rahele Mehraeen, Emadoddin Moudi, Ali Rostami, Mohammad Barary, Akram Hosseini, Ali Bijani, Mostafa Javanian

**Affiliations:** ^1^Infectious Diseases and Tropical Medicine Research Center, Health Research Institute, Babol University of Medical Sciences, Babol, Iran; ^2^Department of Radiology, School of Medicine, Babol University of Medical Sciences, Babol, Iran; ^3^Clinical Research Development Center, Shahid Beheshti Hospital, Babol University of Medical Sciences, Babol, Iran; ^4^Student Research Committee, Babol University of Medical Sciences, Babol, Iran; ^5^Department of Pathology, School of Medicine, Babol University of Medical Sciences, Babol, Iran; ^6^Social Determinants of Health Research Center, Health Research Institute, Babol University of Medical Sciences, Babol, Iran

## Abstract

While some biomolecules have been explored to identify potential biomarkers for the prognosis of COVID-19 patients, there is no reliable prognostic indicator of the disease progression and severity. We aimed to evaluate the ability of the C-reactive protein (CRP) to predict COVID-19 infection outcome. This retrospective study was conducted on 429 patients diagnosed with COVID-19 between March 30, 2020, and April 30, 2020. The study population was divided into severe (*n* = 175) and nonsevere cases (*n* = 254). Data on demographic characteristics, clinical features, and laboratory findings on admission were collected. The proportion of patients with increased CRP levels was significantly higher in severe cases than in nonsevere patients. Analysis of the receiver operating characteristic (ROC) curve found that CRP could be used as an independent factor in predicting the severity of COVID-19. Also, patients with CRP >64.75 mg/L were more likely to have severe complications. In conclusion, CRP serum levels can predict the severity and progression of illness in patients with COVID-19.

## 1. Introduction

Since December 2019, a new type of coronavirus called severe acute respiratory syndrome coronavirus 2 (SARS-CoV-2), causing coronavirus disease 2019 (COVID-19), has been identified in China [1]. The COVID-19 pandemic then spread quickly around the world [2, 3]. By March 30, 2021, 127,349,248 confirmed cases of COVID-19, including 2,787,593 deaths, were reported to the World Health Organization (WHO) [4]. The rapid spread of the SARS-CoV-2, rapid changes in clinical features, and increased mortality have become the world's biggest concern. Furthermore, there are no reliable prognostic indicators for predicting disease severity and progression. Recognizing markers of disease severity may thus profoundly help to detect at-risk patients. Recently, some studies have reported that C-reactive protein (CRP) levels can be used in the early diagnosis of pneumonia and that higher CRP levels were associated with severe pneumonia [5].

Hence, the current study aims to evaluate the correlation between CRP levels and disease progression to provide a reference for the clinical management of COVID-19 patients.

## 2. Materials and Methods

### 2.1. Study Design, Participants, and Definition

The present retrospective study was approved by the Ethics Committee of Babol University of Medical Sciences (code: IR.MUBABOL.REC.1399.041). The three affiliated hospitals of Babol University of Medical Sciences have been designated to treat patients with COVID-19. A total of 429 adult cases were confirmed at these centers from March 30 to April 30, 2020. All patients with COVID-19 who enrolled in the recent study were diagnosed according to the WHO interim guidance for COVID-19 (6th edition) [6]. In other words, all patients with the physician- and laboratory-confirmed (positive nasopharyngeal/throat swab specimens by reverse transcription-polymerase chain reaction (RT-PCR)) COVID-19 infection were included, while suspected cases with similar clinical symptoms were excluded. All cases were monitored using the clinical data collected until March 30, 2020. One of the following criteria was used to determine severe COVID-19 illness: respiratory rate ≥30 bpm, oxygen saturation ≤93%, arterial oxygen partial pressure (PaO_2_)/oxygen concentration (FiO_2_) ≤300 mm Hg, and intensive care unit (ICU) admission.

### 2.2. Data Collection

Patient medical records were reviewed by an experienced team of clinicians of the Infectious Diseases and Tropical Medicine Research Center of Babol University of Medical Sciences. The data on epidemiological, clinical, laboratory, radiological findings, and outcomes were collected using a data collection checklist from electronic medical records. Moreover, recorded patient data, such as demographic characteristics, past medical history (PMH), underlying medical conditions, symptoms, and signs, were collected.

### 2.3. Statistical Analysis

The statistical data were analyzed using SPSS version 16.0 (IBM, Chicago, IL, USA). Continuous and categorical variables were presented as median (IQR) and *n* (%), respectively. Mann–Whitney *U*-test and Student's *t*-test were used to compare continuous and categorical variables. The predictive value of the CRP was evaluated by measuring the area under the receiver operating characteristic curve (AUC). The optimal threshold value was obtained by calculating the Youden index. A multivariate Cox proportional risk model was used to determine predictive factors for disease risk.

## 3. Results

There were 429 patients with COVID-19 in our study. Of these, 175 patients (40.8%) were assigned to the severe group, while 254 patients (59.2%) were allocated to the nonsevere group. The demographic and clinical characteristics of the patients are summarized in Table 1. The mean age was 57.21 ± 16.18 years, with a range of 16–99 years. The average age was higher in the severe group than in the nonsevere group (*P*=0.111). One hundred and eighty-six patients (43.4%) were female. The severity ratio for males was higher than for females, but this difference was not significant (*P*=0.122). The median duration from illness onset to discharge was seven days. Overall, dyspnea (72.5%) was the most common initial symptom, followed by fever (61.3%) and dry cough (57.6%). However, there was no significant difference in the symptoms ratio between the two groups.

Moreover, nearly half of the patients (213, 49.7%) had comorbidities, such as diabetes (27.7%), cardiovascular disease (24.9%), and hypertension (22.8%). Sixty-two patients (14.5%) had a complication, including the occurrence of acute respiratory distress syndrome (ARDS) (18 (10.3%) vs. 0 (0), *P*=0.101), acute heart failure (9 (5.1%) vs. 19 (7.5%), *P*=0.335), and arrhythmia (8 (4.6%) vs. 11 (4.3%), *P*=0.335) (Table 1). Laboratory findings of the patients are presented in Table 2. Median levels of lymphocyte count, erythrocyte sedimentation rate (ESR), C-reactive protein (CRP), and lactate dehydrogenase (LDH) were not in the normal range for the severe group. These patients had a significantly lower lymphocyte count (MD: 12 vs. MD: 22.7, *P* < 0.001) and a significantly higher ESR (MD: 57.5 vs. MD: 40, *P*=0.005), CRP (MD: 97 vs. MD: 50, *P* < 0.001), and LDH (MD: 783.5 vs. MD: 459, *P* < 0.001) levels compared to the nonsevere group.

**Figure 1:**
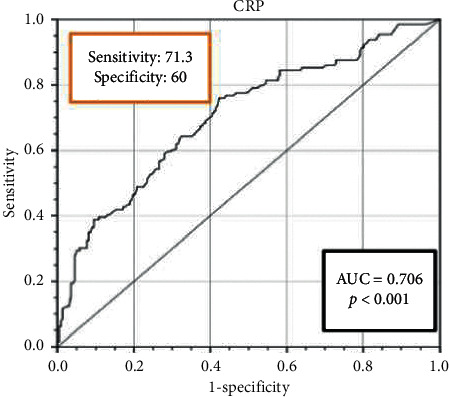
Receiver operating characteristic (ROC) curves of C-reactive protein for predicting the disease severity in COVID-19 patients.

Furthermore, analysis of the ROC curve illustrated an 0.706 area under the curve (AUC) for CRP levels as a predictor of disease severity (95% CI: 0.649–0.764; *P* < 0.001). The AUC of this biomarker indicated a high diagnostic value for clinical severity, with the optimal threshold value being 64.75 mg/L with a sensitivity of 71.32% and a specificity of 60% (Table 3 and Figure 1). We reclassified patients into two groups according to the optimal CRP threshold (cutoff: 64.75 mg/L). The proportion of severe patients with a CRP level higher than the optimal threshold was significantly different from that of a lower CRP level (*P* < 0.001).

The univariate analysis used in the logistic model indicated the severity was associated with hospital admission (OR: 1.166; 95% CI: 1.119–1.216; *P* < 0.001), BUN (OR: 1.027; 95% CI: 1.014–1.040; *P* < 0.001), lymphocyte count (OR: 0.917; 95% CI: 0.895–0.939; *P* < 0.001), and CRP level (OR: 3.647 95% CI: 2.288–5.813; *P* < 0.001). As determined by multivariate analysis, hospital admission (OR: 1.185; 95% CI: 1.086–1.293; *P* < 0.001) and CRP level (OR: 3.826; 95% CI: 1.166–12.560; *P*=0.027) were significantly associated with severity, so that the patients with a CRP level >64.75 mg/L were more likely to develop the severe form of the disease (Table 4).

## 4. Discussion

In the present retrospective study, the clinical characteristics of severe COVID-19 patients were compared with those of nonsevere patients and analyzed the possible factors associated with disease progression and severity. Furthermore, the prognostic value of the CRP in the progression of COVID-19 cases has been revealed. The current study evaluated the association between CRP and COVID-19 infection, and the findings indicated that a patient with a CRP level >64.75 mg/L was more likely to develop the severe form of the disease. In other words, ROC analysis confirmed CRP as a valuable predictor of COVID-19 progression and severity.

In response to infections, the liver synthesizes significant quantities of acute-phase proteins (APPs), such as CRP [7, 8]. This acute inflammatory protein is a highly sensitive biomarker for inflammation, tissue damage, and infection [9]. It has been shown that CRP levels are correlated with levels of inflammation [10]. CRP levels can promote phagocytosis and activate the complement system [11]. In other words, CRP binds to microorganisms and promotes their removal through phagocytosis [12].

Moreover, the serum CRP levels increase during inflammatory responses. As shown previously, this biomarker may be raised by viral or bacterial infections. It is important to note that CRP levels were significantly increased in bacterial infections than in viral infections [13]. The current study revealed significantly higher CRP levels in severe cases than in nonsevere patients suggesting that the CRP level may be a biomarker of disease severity and progression in patients with COVID-19. Liu et al. reported that more severe cases infected with COVID-19 expressed significantly higher CRP levels than nonsevere patients [14]. Qin et al. observed higher CRP levels in severe COVID-19 patients than in nonsevere cases, suggesting that this biomarker can be monitored to evaluate disease progression [15]. Sahu et al. performed a meta-analysis to assess CRP levels as a potential biomarker of the COVID-19 prognosis. Their results indicated that CRP concentrations remain high in expired patients and could be a promising biomarker for assessing mortality [16]. Also, some studies showed that some frequent complications in severe and expired COVID-19 patients, such as shock, ARDS, acute kidney injury, and acute cardiac injury, were correlated with higher CRP levels [17].

## 5. Limitations and Future Suggestions

The current study encompasses a short sample size. Thus, it may lack generalizability. Furthermore, the retrospective nature of this study and the consequent missing clinical data was another limitation. Therefore, subsequent clinical studies with larger sample sizes and multiple CRP level measurements, especially at different treatment times, should be performed to confirm our findings.

## 6. Conclusions

Our findings suggest that serum CRP levels could be used as an essential indicator of the progression and the severity of COVID-19. Also, the present study suggests that patients with higher CRP levels should be carefully monitored throughout their disease course.

## Figures and Tables

**Table 1:** Demographic and baseline characteristics of patients with COVID-19.

		All patients (*n* = 429)	Severe (*n* = 175)	Nonsevere (*n* = 254)	*P* value
Age, mean ± SD (range)		57.21 ± 16.18 (16–99)	58.75 ± 15.88 (22–97)	56.18 ± 16.32 (16–99)	0.111

Sex (%)	Male	241 (56.4)	106 (60.9)	135 (53.4)	0.122
	Female	186 (43.6)	68 (39.1)	118 (46.6)	

Hospitalization (days) (range)		7 (1–37)	10 (1–37)	6 (1–31)	<0.001

Clinical symptoms (%)	Fever	263 (61.3)	105 (60)	158 (62.2)	0.645
	Fatigue	161 (37.5)	70 (40)	91 (35.8)	0.380
	Headache	76 (17.7)	31 (17.7)	45 (17.7)	1.000
	Dry cough	247 (57.6)	98 (56)	149 (58.7)	0.584
	Sore throat	36 (8.4)	19 (10.9)	17 (6.7)	0.126
	Expectoration	83 (19.3)	34 (19.4)	49 (19.3)	0.972
	Hemoptysis	7 (1.6)	3 (1.7)	4 (1.6)	0.911
	Chest pain	61 (14.2)	22 (12.6)	39 (15.4)	0.417
	Dyspnea	311 (72.5)	126 (72)	69 (72.8)	0.849
	Nausea	81 (18.9)	37 (21.1)	44 (17.3)	0.320
	Diarrhea	25 (5.8)	12 (6.9)	13 (5.1)	0.450
	Constipation	24 (5.6)	9 (5.1)	15 (5.9)	0.736
	Anorexia	118 (27.5)	53 (30.3)	65 (25.6)	0.284
	Arthralgia	48 (11.2)	20 (11.4)	28 (11)	0.896
	Stomach ache	39 (9.1)	16 (9.1)	23 (9.1)	0.975
	Dizziness	54 (12.6)	21 (12)	33 (13)	0.761
	Loss of smell and taste	18 (4.2)	9 (5.1)	9 (3.5)	0.417

Comorbidities (%)	Hypertension	98 (22.8)	32 (18.3)	66 (26)	0.062
	Cardiovascular disease	107 (24.9)	38 (21.7)	185 (27.2)	0.200
	Diabetes	119 (27.7)	49 (28)	70 (27.6)	0.920
	Cancer	10 (2.3)	3 (1.7)	7 (2.8)	0.482
	Chronic liver disease	5 (1.2)	3 (1.7)	2 (0.8)	0.379
	Chronic kidney disease	17 (4)	2 (1.1)	15 (5.9)	0.013
	Brain disease	9 (2.1)	6 (3.4)	3 (1.2)	0.110
	COPD	14 (3.3)	7 (4)	7 (2.8)	0.476

Complications (%)	Nosocomial pneumonia	8 (1.9)	2 (1.1)	6 (2.4)	0.359
	Urinary tract infection	6 (1.4)	2 (1.1)	4 (1.6)	0.708
	Shock	5 (1.2)	5 (2.8)	0 (0)	0.073
	Acute heart failure	28 (6.5)	9 (5.1)	19 (7.5)	0.335
	Arrhythmia	19 (4.4)	8 (4.6)	11 (4.3)	0.905
	ARDS	18 (4.2)	18 (10.3)	0 (0)	0.101
	Acute kidney failure	1 (0.4)	0 (0)	1 (0.4)	0.406

Abbreviations: ARDS, acute respiratory distress syndrome; COPD, chronic obstructive pulmonary disease.

**Table 2:** Laboratory findings of severe and nonsevere COVID-19 patients.

	Normal range	Severe (*n* = 175)	Nonsevere (*n* = 254)	*P* value
White blood cell count, median (range) (×10_3_/*μ*L)	4,500–11,000	9,100 (1,700–42,300)	6,500 (2,100–157,000)	<0.001
Lymphocytes, %, median (range)	26–46	12 (2–36)	22.7 (3–75)	<0.001
Hemoglobin, median (range) (g/dL)	13.5–18	11.95 (6–21)	12.5 (5.3–22.3)	0.032
Erythrocyte sedimentation rate, median (range) (mm/h)	0–15	57.5 (3–128)	40 (2–140)	0.005
C-reactive protein, median (range) (mg/L)	0–10	97 (1–440)	50 (4–392)	<0.001
Platelet count, median (range) (count/*µ*L)	140,000–450,000	197,000 (42,000–568,000)	197,000 (8,000–108,1000)	0.839
Blood urea nitrogen, median (range) (mg/dL)	10–20	19 (5–158.2)	15 (4–150)	<0.001
Creatinine, median (range) (mg/dL)	0.7–1.4	1 (0.5–7.4)	1 (0.5–7.3)	0.360
Lactate dehydrogenase, median (range) (U/L)	140–280	783.5 (146–2,436)	459 (30–2,500)	<0.001
Sodium, median (range) (mEq/L)	135–145	135 (117–146)	136 (120–152)	0.144
Potassium, median (range) (mEq/L)	3.7–5.2	4.1 (3–8.2)	4.1 (2.9–5.5)	0.961

**Table 3:** The area under the ROC curve (AUC) and the optimal cutoff value of CRP.

AUC	Optimal cutoff value (mg/L)	Sensitivity (%)	Specificity (%)	Predictive value		Likelihood ratio	
				Positive (%)	Negative (%)	Positive	Negative
0.706	64.75	71.32	60	78.11	50.55	0.48	1.76

Abbreviations: AUC, area under the curve.

**Table 4:** Univariate and multivariate analyses of the logistic regression model.

Variable	Univariate		Multivariate	
	Odds ratio (95% CI)	*P* value	Odds ratio (95% CI)	*P* value
Age	1.010 (0.998–1.022)	0.112	1.003 (0.963–1.030)	0.762
Hospital admission (days)	1.166 (1.119–1.216)	<0.001	1.185 (1.086–1.293)	<0.001
Lymphocytes	0.917 (0.895–0.939)	<0.001	0.990 (0.934–1.050)	0.741
BUN	1.027 (1.014–1.040)	<0.001	1.045 (0.994–1.097)	0.082
Diabetes	1.022 (0.665–1.571)	0.920	2.543 (0.809–7.998)	0.110
CRP	3.647 (2.288–5.813)	<0.001	3.826 (1.166–12.560)	0.027

ARDS, acute respiratory distress syndrome; COPD, chronic obstructive pulmonary disease; BUN, blood urea nitrogen; CRP, C-reactive protein; CI, confidence interval.

## Data Availability

All data are available upon reasonable request to the corresponding author.
